# The Association Between Climate Change Awareness, Climate Anxiety, and Carbon Footprint Awareness Among Nursing Students

**DOI:** 10.1002/nop2.70692

**Published:** 2026-07-12

**Authors:** Tuğba Şahin Tokatlioğlu, Beyzanur İşbay Aydemir, Seda Karakaya Çataldaş

**Affiliations:** ^1^ Department of Nursing, Faculty of Health Sciences Istanbul Aydın University İstanbul Turkey; ^2^ Department of Psychiatric and Mental Health Nursing Koc University, School of Nursing İstanbul Turkey

## Abstract

**Aim:**

To examine the association between climate change awareness, climate anxiety, and carbon footprint awareness among nursing students.

**Design:**

A cross‐sectional, descriptive, and correlational study.

**Methods:**

The study was conducted with 715 nursing students using self‐administered questionnaires to assess their climate change awareness, climate change anxiety, and carbon footprint awareness. Sociodemographic information was also collected. Associations among variables were examined using correlation analysis, and associated factors related to carbon footprint awareness were evaluated through hierarchical regression analysis.

**Results:**

Carbon footprint awareness was positively associated with both climate change awareness and climate change anxiety, with anxiety showing a stronger association. Psychosocial variables explained variance in carbon footprint awareness more strongly than sociodemographic characteristics (*R* ^2^ = 0.13). Subgroup analyses showed no significant differences in carbon footprint scores according to academic year, previous awareness of the term ‘carbon footprint’, or place of residence. Climate change awareness was higher among students who had previously heard the term ‘carbon footprint’ (*p* = 0.013), whereas climate change anxiety differed significantly by academic year (*p* < 0.001).

**Conclusion:**

The findings suggest that carbon footprint awareness among nursing students is more closely related to psychosocial processes than sociodemographic characteristics. Climate change anxiety, in particular, appears to be an important factor associated with environmentally related awareness. Additional subgroup analyses showed no significant differences in carbon footprint scores across subgroups, whereas climate change awareness and anxiety varied according to certain subgroup characteristics. However, the findings should be interpreted cautiously given the cross‐sectional design and the relatively low explained variance of the model.

**Implications for Nursing Practice:**

Integrating carbon footprint literacy and climate change into nursing curricula, considering peer education models, and promoting sustainable practices such as reducing the use of single‐use materials during clinical education may contribute to increasing environmental awareness and supporting environmentally responsible nursing practice among nursing students.

**Reporting Method:**

This study adhered to the Strengthening the Reporting of Observational Studies in Epidemiology (STROBE) guideline.

**Patient or Public Contribution:**

No patient or public contribution.

## 1 Introduction

In recent years, the impacts of climate change on ecosystems and human health have intensified, rendering it a global climate crisis for both current and future generations (Romanello et al. 2022; WHO 2021). Direct environmental changes such as rising temperatures, melting glaciers, and sea level rise have led to severe adverse consequences for agriculture, water resources, and natural ecosystems (Legg 2021). At the same time, the health‐related effects of climate change have become increasingly pronounced, particularly among vulnerable populations, including students (Haines et al. 2009; Sahin‐Bayindir et al. 2024).

One of the key factors contributing to this crisis is carbon dioxide (CO_2_) emissions, which accelerate global warming by trapping heat in the atmosphere and disrupting the climate balance (Klingelhöfer et al. 2020). Notably, compared to pre‐industrial levels (1850–1900), the global average temperature has increased by around 1.4°C, and the exceptional temperatures observed during 2023–2025 marked the first three‐year period on record in which the global average temperature exceeded 1.5°C above the pre‐industrial level (WMO 2026; Aalto et al. 2026). Therefore, reducing carbon emissions and lowering individuals' carbon footprints are critical to mitigating the impacts of climate change and are considered an urgent public health priority, particularly within the scope of the United Nations Sustainable Development Goals, especially SDG 13 (Álvarez‐Nieto et al. 2022).

### 1.1 Climate Change, Health, and Psychosocial Impacts

Climate change not only affects physical health but also generates psychosocial consequences, including anxiety, stress, and reduced quality of life through mechanisms such as heatwaves, air pollution, and other environmental stressors (Zhao et al. 2022; Luber and Lemery 2015). Climate change awareness refers to the understanding and recognition of the causes and impacts of climate change (Charlson et al. 2021). In this context, one of the psychosocial outcomes increasingly addressed by researchers is eco‐anxiety, which encompasses fears about environmental degradation and its effects on the future (Clayton and Karazsia 2020; Feather and Williams 2021; Hogg et al. 2023; Lutz et al. 2023; Ojala et al. 2021). Climate anxiety, on the other hand, is considered a more specific form of eco‐anxiety that refers directly to worries and concerns related to climate change and its anticipated impacts (Boluda‐Verdú et al. 2022). Research shows that heightened awareness of climate issues often leads to increased anxiety about ecological outcomes, highlighting a complex interplay between knowledge and emotional response in the context of climate crises (Clayton et al. 2017; Charlson et al. 2021). In parallel with increasing awareness of climate‐related environmental problems, the concept of carbon footprint awareness has also gained importance, referring to individuals' awareness of the environmental impacts of daily activities and behaviours that contribute to greenhouse gas emissions and climate change (Wiedmann and Minx 2008).

However, conceptual clarity regarding the relationships among climate change awareness, eco‐anxiety, and climate anxiety has not yet been fully established. In other words, there is no clear consensus in the literature on whether individuals experiencing eco‐anxiety are negatively affected in terms of psychological distress or whether this anxiety fosters determination and leads to pro‐environmental behaviours (Hogg et al. 2023; Ogunbode et al. 2023; Pihkala 2020). Indeed, a study conducted among nursing students reported that higher levels of eco‐anxiety were associated with an increased tendency to engage in environmentally friendly behaviours (Er et al. 2024). Consequently, further research and empirical evidence are needed to clarify these concepts (Coffey et al. 2021).

### 1.2 Nursing, Climate Change, and Professional Responsibility

Nurses, who play a crucial role in promoting public health and well‐being, are reportedly at the forefront of efforts to address climate change (Evans‐Agnew and Aguilera 2023). Nursing students, as future healthcare professionals, occupy a critical position in raising awareness among individuals and communities and preparing them for the impacts of climate change (Amerson et al. 2022; Dağlı et al. 2024).

In this regard, the nursing profession has demonstrated early leadership in this field. The American Nurses Association (ANA) became one of the first healthcare organizations to take action on climate change, adopting the Climate Change Resolution in 2008 through its House of Delegates. Furthermore, the ANA's report, entitled ‘The Role of Nurses in Addressing Global Climate Change, Climate Justice, and Health’, provides detailed guidance on interventions and adaptation strategies that nurses can implement to address health issues arising from climate change (ANA 2023).

### 1.3 Climate Awareness, Nursing Education, and Sustainability

Given the critical role of the nursing profession, education plays a pivotal role in ensuring that these issues are adequately addressed during training. Education is recognized as a fundamental tool both for achieving the Sustainable Development Goals and for combating climate change (Shaw et al. 2021). As nurses constitute the largest segment of the global healthcare workforce (WHO 2022), equipping them with sustainability‐oriented knowledge and skills is crucial for enabling them to take an active and effective role in this process (International Council of Nurses 2018).

Accordingly, nursing education should extend beyond the development of clinical competencies to include curricula that incorporate sustainability and environmental awareness (Goodman and East 2013). Climate awareness encompasses components such as recognizing risks, acquiring knowledge, and developing sustainable lifestyle behaviours (Amerson et al. 2022; İlaslan and Şahin Orak 2024). It has been suggested that such awareness contributes positively to students' psychological well‐being and quality of life.

### 1.4 Theoretical Framework and Research Aim

Ecological Systems Theory and the Social Determinants of Health Model emphasize that environmental conditions and individual awareness jointly shape health outcomes (Bronfenbrenner 1979; Marmot 2005), thereby supporting the importance of examining the effects of climate change awareness and anxiety on students' environmental behaviours. Therefore, determining the extent to which climate change awareness and climate‐related anxiety are associated with carbon footprint awareness among university students is crucial for environmental sustainability and public health.

Existing studies have mainly focused on the knowledge, perceptions, awareness, attitudes, and educational needs of nursing students regarding climate change and sustainability. A systematic review by Shah et al. (2024) found that nursing students' knowledge of climate change and its health‐related impacts, with emphasis on demographic and educational correlates of knowledge levels. Similarly, an integrative review by Aronsson et al. (2024) examined awareness, attitudes, and sustainability‐related actions among nursing students and educators. The scoping review by Bérubé et al. (2024) explored nurses' and nursing students' perceptions of climate change and identified barriers to climate‐related practice. In addition, the scoping review by Tiitta et al. (2024) focused primarily on teaching strategies, educational interventions, and nursing students' perceptions of climate change education in nursing curricula. More recently, Kaşıkçı et al. (2025) explored nursing students' awareness and perceptions regarding carbon footprint and environmental sustainability through a qualitative approach, whereas Xu et al. (2025) examined nursing students' support for environmental measures in healthcare services and the factors associated with this support. Consequently, these studies largely provided descriptive evidence concerning knowledge, perceptions, competencies, and educational experiences. However, the relationship between climate change awareness, climate anxiety, and carbon footprint awareness among nursing students has not yet been specifically investigated. Thus, this study aimed to examine the association between climate change awareness, climate anxiety, and carbon footprint awareness among nursing students who are expected to play an active role in sustainability, environmental health, and climate‐related public health initiatives.

## 2 Methods

### 2.1 Design

This study was conducted to determine the association between climate change awareness, climate anxiety, and carbon footprint awareness among nursing students, employing a cross‐sectional, descriptive, and correlational design.

### 2.2 Study Setting

The study was conducted during the fall semester of the 2025–2026 academic year with undergraduate nursing students enrolled in the Department of Nursing at a foundation university. The fall semester was selected because it represented the beginning of the academic year, before the commencement of courses, and because no climate change‐related course had yet been included in the students' curriculum during the study period. This study adhered to the Strengthening the Reporting of Observational Studies in Epidemiology (STROBE) guideline (Babaoğlu et al. 2021) (see File S1).

### 2.3 Participants

A convenience sampling approach was used, and students who met the inclusion criteria and voluntarily agreed to participate were included in the study. As no climate change‐related courses had yet been included in the curriculum during the study period, students from all academic years were included in the study without any distinction based on academic year. However, information on academic year was collected through the sociodemographic form to allow for the evaluation of potential differences across academic years. The survey link was shared with all students who met the inclusion criteria, including first‐, second‐, third‐, and fourth‐year nursing students. Faculty members involved in the study were employed in the Department of Nursing and taught courses to these students, which facilitated the distribution of the survey link. In addition, the survey link was shared through a WhatsApp group including student representatives from all academic years, and the representatives further disseminated the link to their respective class groups.

#### 2.3.1 Inclusion Criteria

- Being enrolled as a student in the Department of Nursing.
- Voluntarily agreeing to participate in the study.
- Being 18 years of age or older.
- Having sufficient proficiency in Turkish to read and understand the scales.
- Having an active student status at the university during the data collection period.

#### 2.3.2 Exclusion Criteria

- Inability to complete the questionnaire form containing the relevant scales.

### 2.4 Sample Size

The total number of nursing students enrolled in the Department of Nursing at the Faculty of Health Sciences at a foundation university during the study period was 746. The sample size was calculated based on the total number of nursing students enrolled in the faculty during the study period (*N* = 746). The required sample size was calculated using Raosoft, a sample size calculation tool for known populations, based on a 1% margin of error and a 99% confidence level, resulting in a minimum required sample of 714 students. A high confidence level was preferred to increase the representativeness of the sample and reduce sampling error. To account for a potential 1% data loss, the target sample size was increased to 721 students. Of the total population, 721 students voluntarily agreed to participate in the study. However, six students were excluded from the final sample due to incorrect responses to the attention‐check (control) item included in the questionnaire. Consequently, the study was completed with a total of 715 students.

### 2.5 Data Collection

The study was conducted at a single center, namely the Department of Nursing at the Faculty of Health Sciences, a foundation university. Data were collected using an online questionnaire created via Google Forms. Prior to data collection, the purpose and scope of the study were explained to all nursing students enrolled in the faculty via email and face‐to‐face communication, and the questionnaire link was distributed to eligible participants. Students who voluntarily agreed to participate provided electronic informed consent before completing the questionnaire. To enhance data quality and response reliability, an attention‐check (control) item was included in the questionnaire, instructing participants to select ‘10’ when responding to the item; students who failed to answer this item correctly were excluded from the study. The average completion time for the questionnaire was approximately 15–20 min.

### 2.6 Measurements

Data were collected using the sociodemographic characteristics form, an awareness scale of university students about global climate change, a carbon footprint awareness scale, and a climate change anxiety scale. Permissions for the scales used in the study were obtained via e‐mail from the authors who conducted the Turkish validity and reliability studies of the instruments. The scales were used in the study after the necessary permissions had been obtained.

#### 2.6.1 Sociodemographic Characteristics Form

The sociodemographic data form used in this study was designed to collect information on the participants' basic individual and familial characteristics. The form included questions regarding students' age and year of study, the type of settlement in which they had lived for the most extended period (village/town, district, city center, metropolitan area), and their perceptions of their family's income level. In addition, information was collected on the educational levels of the participants' parents and the type of family they lived in (nuclear, extended, or single‐parent family) (Dağlı et al. 2024; Sengul et al. 2025). Finally, participants were asked whether they had previously heard the term ‘carbon footprint’ in order to assess their level of awareness of environmental concepts. In addition, an attention‐check item was included in the form. Participants were instructed to select ‘10’ when responding to this item to ensure that they had carefully read the question.

#### 2.6.2 Awareness Scale of University Students About Global Climate Change

The Awareness Scale of University Students About Global Climate Change, developed by Deniz et al. (2021), was used to determine the levels of awareness among university students regarding global climate change (Deniz et al. 2021). The scale consists of 21 items and is rated on a 5‐point Likert scale ranging from 1 (Strongly Disagree) to 5 (Strongly Agree), with no reverse‐coded items. The minimum possible score obtainable from the scale is 21, and the maximum score is 105. Awareness levels are interpreted as low for mean scores between 1.00 and 2.33, moderate for scores between 2.34 and 3.66, and high for scores between 3.67 and 5.00. All dimensions of the scale are summable, and higher scores indicate a higher level of awareness of climate change. Permission to use the scale was obtained, and it is recognized in the literature as a valid and reliable measurement tool. In the original study, the Cronbach's alpha coefficient of the scale was reported as 0.826. In the present study, the scale demonstrated excellent internal consistency, with a Cronbach's alpha coefficient of 0.923.

#### 2.6.3 Carbon Footprint Awareness Scale

The scale is widely used in the literature as a valid and reliable instrument for assessing awareness regarding carbon footprint. The scale was developed by Üstgörül et al. (2024) and consists of 19 items rated on a 5‐point Likert scale ranging from 1 (Never) to 5 (Always). Items 2, 6, 12, and 13 are reverse‐coded. Total scores obtained from the scale range from 19 to 95, with a cutoff point of 57 (Üstgörül et al. 2024). Scores of 57 and above indicate high awareness of carbon footprint, whereas scores below 57 indicate low awareness of carbon footprint. The Carbon Footprint Awareness Scale was used to assess students' self‐reported awareness and behavioural practices related to reducing their carbon footprint. Although the scale is titled as an awareness scale, its items mainly reflect everyday behavioural preferences and practices in domains such as transportation, energy use, electricity consumption, food consumption, and waste management, as reflected in items such as ‘I prefer using public transportation for long‐distance travel’. All scale items are provided in File S1. In the original study, the scale demonstrated good internal consistency, with a Cronbach's alpha coefficient of 0.86. In the present study, the scale demonstrated moderate internal consistency based on Cronbach's alpha (*α* = 0.67), whereas McDonald's omega coefficient indicated excellent internal consistency (*ω* = 0.96).

#### 2.6.4 Climate Change Anxiety Scale

The scale, originally titled the ‘Psychometric Properties of the Climate Change Worry Scale’, was developed by Stewart (2021), and its Turkish adaptation was conducted by Özbay and Alcı (2021). The Climate Change Worry Scale consists of 10 items and is rated on a 5‐point Likert scale ranging from 1 (Never), 2 (Rarely), 3 (Sometimes), 4 (Often), to 5 (Always). The minimum possible score obtainable from the scale is 10, and the maximum score is 50. In the Turkish adaptation study, the scale demonstrated excellent internal consistency, with a Cronbach's alpha coefficient of 0.98. In the present study, the Cronbach's alpha coefficient was calculated as 0.87, indicating good internal consistency.

### 2.7 Statistical Analysis

Statistical analyses were performed using IBM SPSS Statistics for Windows, Version 28.0 (IBM Corp., Armonk, NY, USA). Descriptive statistics were used to summarize the data. Normality was assessed using skewness and kurtosis values (±1.5), and parametric analyses were applied. Pearson correlation analysis was conducted to examine relationships between carbon footprint scores, climate change awareness, and climate change anxiety. Hierarchical linear regression analysis was used to identify predictors of carbon footprint scores. Regression assumptions were met, and multicollinearity was not detected (VIF < 5). Results were reported using standardized beta coefficients (*β*), *R* ^2^, adjusted *R* ^2^, and Δ*R* ^2^. A two‐tailed *p*‐value < 0.05 was considered statistically significant.

### 2.8 Ethical Considerations

Ethical approval for this study was obtained from the Koç University Social and Human Sciences Ethics Committee (approval number 2025.555.IRB3.219, 2 December 2025). Institutional permission was also obtained from the foundation university where the data were collected. In addition, permission to use the instruments included in the study was obtained via e‐mail from the authors who conducted the Turkish validity and reliability studies of the scales. Throughout the study, strict adherence was maintained to the ethical principles outlined in the Declaration of Helsinki, including confidentiality, beneficence, respect, justice, and non‐maleficence. Written informed consent was obtained from all participants prior to the commencement of the study. Participants were clearly informed that they had the right to withdraw from the study or terminate their participation at any time without penalty. The confidentiality of participant information was one of the highest priorities. All collected data were used solely for research purposes and kept strictly confidential. No personally identifiable information was included in the study reports.

## 3 Results

A total of 715 participants were included in this study. As shown in Table 1, the mean age of the participants was 20.86 years (SD = 2.14). Most participants were in their second year of study (40.8%), followed by first‐year students (28.5%). The vast majority of participants reported living in a nuclear family (67.1%) and indicated that their family income was lower than their expenses (52.9%). Regarding parental education, most mothers had completed secondary school (64.5%), while fathers most commonly reported a high school level of education (41.5%). Nearly half of the participants resided in metropolitan cities (48.8%). Regarding carbon footprint awareness, 60.0% of the participants reported having heard of the concept.

**TABLE 1:** Sociodemographic characteristics of the participants (*N* = 715).

Variable	Category	*n*	%
Age, M (SD)	20.86 (2.14)		
Class year	1st year	204	28.5
	2nd year	292	40.8
	3rd year	106	14.8
	4th year	113	15.8
Family type	Nuclear family	480	67.1
	Extended family	195	27.3
	Single‐parent family	40	5.6
Family income status	Income less than expenses	378	52.9
	Income equal to expenses	256	35.8
	Income more than expenses	81	11.3
Education of mother	Primary school	107	15.0
	Secondary school	461	64.5
	High school	147	20.6
Education of father	Primary school	112	15.7
	Secondary school	206	28.8
	High school	297	41.5
	University and above	100	14.0
Place of residence	Village	88	12.3
	District	126	17.6
	Metropolitan city	349	48.8
	Province	152	21.3
Carbon footprint awareness	Yes	429	60.0
	No	286	40.0

As shown in Table 2, Pearson correlation analyses indicated that carbon footprint scores were positively associated with both climate change awareness (*r* = 0.24, 95% CI [0.16, 0.33], *p* < 0.01) and climate change anxiety (*r* = 0.26, 95% CI [0.18, 0.33], *p* < 0.01), reflecting small‐to‐moderate effect sizes. Climate change awareness and climate change anxiety were also positively correlated (*r* = 0.22, 95% CI [0.13, 0.30], *p* < 0.01), indicating a small effect size.

**TABLE 2:** Pearson correlation among carbon footprint, climate change awareness, and climate change anxiety.

Variable	1	2	3
1. Carbon footprint	—		
2. Climate change awareness	0.24** [0.16, 0.33]	—	
3. Climate change anxiety	0.26** [0.18, 0.33]	0.22** [0.13, 0.30]	—

*Note:* Pearson correlation coefficients (*r*) with 95% confidence intervals in brackets are presented. Correlation coefficients represent effect sizes. Carbon footprint refers to the total carbon footprint score. KİDFÖ = Climate Change Awareness Scale; İDKÖ = Climate Change Anxiety Scale. Sample size ranged from *N* = 713 to 715 due to missing data. Bootstrap confidence intervals are based on 5000 samples.

** *p* < 0.01 (two‐tailed).

As shown in Table 3, hierarchical linear regression analysis was performed to examine the predictors of carbon footprint scores.

**TABLE 3:** Hierarchical linear regression models predicting carbon footprint.

Dependent variable: total carbon footprint score					
Predictor	Model 1 *β*	Model 2 *β*	*B* (Model 2)	95% CI for *B*	*p*
Age	−0.012	−0.020	−0.078	[−0.378, 0.222]	0.611
1st year (ref = 2nd year)	0.022	0.056	1.034	[−0.429, 2.496]	0.166
3rd year	0.030	0.078	1.847	[−0.002, 3.696]	0.050
4th year	−0.035	0.051	1.182	[−0.746, 3.111]	0.229
Extended family	0.014	0.007	0.125	[−1.249, 1.500]	0.858
Divided family	0.017	0.018	0.668	[−1.924, 3.260]	0.613
Income = expenses	0.051	0.057	0.998	[−0.300, 2.297]	0.132
Income > expenses	−0.026	−0.028	−0.749	[−2.735, 1.236]	0.459
Mother education—primary	−0.065	−0.046	−1.071	[−2.785, 0.643]	0.220
Mother education—high school	−0.050	−0.069	−1.439	[−2.952, 0.075]	0.062
Father education—primary	0.011	0.026	0.602	[−1.242, 2.446]	0.522
Father education—middle school	0.035	0.026	0.490	[−0.980, 1.960]	0.513
Father education—university+	−0.086*	−0.086*	−2.092	[−3.932, −0.253]	0.026
Village	0.016	−0.009	−0.221	[−2.179, 1.737]	0.825
District	0.022	0.012	0.262	[−1.414, 1.939]	0.759
Province	−0.007	0.006	0.118	[−1.451, 1.687]	0.882
Climate change awareness	—	0.191***	0.098	[0.061, 0.136]	< 0.001
Climate change anxiety	—	0.240***	0.257	[0.175, 0.339]	< 0.001

*Note:* Model fit indices: Model 1: *R* ^2^ = 0.024, Adjusted *R* ^2^ = 0.003, *F* change = 1.15, *p* = 0.305. Model 2: *R* ^2^ = 0.130, Adjusted *R* ^2^ = 0.108, Δ*R* ^2^ = 0.106, *F* change = 42.16, *p* < 0.001. Durbin–Watson = 1.96. Values are standardized regression coefficients (*β*). Reference categories: 2nd year, nuclear family, income < expenses, father education = high school, metropolitan city. *p < 0.05; ***p < 0.001.

In Model 1, which included sociodemographic variables, the overall model was not found statistically significant (*R* ^2^ = 0.024, adjusted *R* ^2^ = 0.003, *F* change = 1.15, *p* = 0.305), indicating that sociodemographic factors did not significantly predict the variance in carbon footprint scores. In this model, having a father with a university‐level degree or above was found to be negatively associated with carbon footprint scores (*β* = −0.086, *B* = −2.081, 95% CI [−4.025, −0.137], *p* = 0.036).

In Model 2, climate change awareness and climate change anxiety were added to the model. This model was found to be significant and explained a modest but statistically significant proportion of the variance in carbon footprint scores (*R* ^2^ = 0.130, adjusted *R* ^2^ = 0.108), with an increase in explained variance compared to Model 1 (Δ*R* ^2^ = 0.106, *p* < 0.001).

Although statistically significant, the model explained a relatively modest proportion of the variance in carbon footprint scores. Therefore, the findings should be interpreted with caution, as a substantial portion of the variance remains unexplained. Both climate change awareness (*β* = 0.191, *B* = 0.098, 95% CI [0.061, 0.136], *p* < 0.001) and climate change anxiety (*β* = 0.240, *B* = 0.257, 95% CI [0.175, 0.339], *p* < 0.001) were found to be significant positive predictors of carbon footprint scores with small‐to‐moderate effect sizes. Among sociodemographic variables, being a third‐year student showed a borderline association with carbon footprint scores (*β* = 0.078, *B* = 1.847, 95% CI [−0.002, 3.696], *p* = 0.050). Therefore, this finding should be interpreted cautiously. In contrast, having a father with a university‐level education or higher was negatively associated with carbon footprint scores (*β* = −0.086, *B* = −2.092, 95% CI [−3.932, −0.253], *p* = 0.026) in the final model.

Additional subgroup analyses were conducted according to academic year, previous awareness of the term ‘carbon footprint’, and place of residence (metropolitan vs. non‐metropolitan). No significant differences were observed in carbon footprint scores across these subgroups. Climate change awareness scores were significantly higher among students who had previously heard the term ‘carbon footprint’ (77.05 ± 16.19 vs. 73.94 ± 16.35, *p* = 0.013). Climate change anxiety differed significantly according to academic year (*F* = 29.091, *p* < 0.001). Post hoc Games–Howell analyses revealed that second‐year students reported significantly higher climate anxiety scores than first‐year (*p* = 0.002), third‐year (*p* < 0.001), and fourth‐year students (*p* < 0.001), whereas fourth‐year students reported significantly lower climate anxiety scores than first‐year (*p* < 0.001) and third‐year students (*p* = 0.009). No significant differences were observed according to place of residence (Table 4).

**TABLE 4:** Subgroup comparisons of carbon footprint scores, climate change awareness, and climate change anxiety.

Characteristic	*n*	Carbon footprint	Climate awareness	Climate anxiety
		Mean ± SD		
Academic year				
1st year	204	67.80 ± 9.00	75.92 ± 18.61	33.92 ± 7.94ᵃ
2nd year	292	67.48 ± 7.07	76.01 ± 14.34	36.40 ± 7.19ᵇ
3rd year	106	67.96 ± 9.29	75.00 ± 15.98	32.16 ± 7.60ᵃ
4th year	113	66.98 ± 9.73	75.84 ± 17.17	29.04 ± 6.89ᶜ
*F* (*p*)		0.321 (0.810)	0.104 (0.957)	29.091 (< 0.001)
Previous awareness of carbon footprint				
Yes	429	68.05 ± 8.08	77.05 ± 16.19	33.64 ± 7.96
No	286	66.84 ± 8.88	73.94 ± 16.35	34.29 ± 7.69
*t* (*p*)		1.851 (0.065)	2.498 (0.013)	−1.078 (0.281)
Residence				
Non‐metropolitan	366	67.65 ± 8.62	76.09 ± 16.15	33.93 ± 7.84
Metropolitan	349	67.47 ± 8.23	75.51 ± 16.50	33.87 ± 7.88
*t* (*p*)		0.277 (0.782)	0.475 (0.635)	0.114 (0.909)

*Note:* Values are presented as mean ± standard deviation. Different superscript letters indicate statistically significant differences according to Games–Howell post hoc analyses. Climate anxiety scores differed significantly across academic years, with second‐year students reporting the highest scores and fourth‐year students reporting the lowest scores.

## 4 Discussion

This study aims to contribute to the literature on environmental sustainability and nursing by examining the association between climate change awareness, climate anxiety, and carbon footprint awareness among nursing students. The findings indicate that both climate change awareness and climate change anxiety are positively associated with carbon footprint, and that these variables explain carbon footprint awareness more strongly than sociodemographic factors. However, the explained variance of the model remained relatively low (*R* ^2^ = 0.13), indicating that other factors not examined in this study may also contribute to carbon footprint awareness such as environmental education, personal lifestyle, social norms, or institutional sustainability practices. These findings suggest that knowledge and emotional responses related to climate change may be associated with self‐reported environmentally responsible tendencies related to carbon footprint among nursing students. Additional subgroup analyses showed that carbon footprint scores did not differ according to academic year, previous awareness of the term ‘carbon footprint’, or place of residence. In contrast, students who had previously heard the term ‘carbon footprint’ reported significantly higher climate change awareness scores, whereas climate change anxiety varied significantly across academic years.

The finding that only 60% of the participants had heard of the concept of carbon footprint and that students who had previously heard the term ‘carbon footprint’ reported higher climate change awareness scores suggests that nursing students possess a basic level of awareness regarding climate change; however, this awareness remains insufficient. Previous studies conducted with nursing students have reported moderate to high levels of awareness regarding reducing ecological footprints including carbon footprint (Atar et al. 2025), as well as above‐moderate levels of support for implementing environmental measures aimed at reducing carbon footprints in healthcare services (Xu et al. 2025). A systematic review by Shah et al. (2024) reported that nursing students' knowledge regarding climate change and its effects on health was generally moderate rather than high. Similarly, Aronsson et al. (2024) noted that although many nursing students were aware of sustainability issues and believed nurses have responsibilities regarding climate change mitigation, some students demonstrated limited awareness and perceived other nursing priorities as more important. These findings suggest that awareness of climate‐related issues among nursing students may be inconsistent and shaped by educational exposure, personal interest, and perceived professional relevance. In addition, Kaşıkçı et al. (2025) found that nursing students' understanding of carbon footprint concepts was largely based on individual perceptions and daily experiences rather than structured environmental education. Taken together, these findings may indicate that although nursing students demonstrate certain environmentally responsible tendencies, their conceptual understanding of climate change and carbon footprint may still be limited. Accordingly, these findings may support the inclusion of more structured content related to climate change and sustainability within nursing education.

The results of the present study are consistent with the existing literature, which demonstrates significant relationships between climate change awareness and increased levels of eco‐anxiety (Mat and Yılmaz 2024). This suggests that heightened climate awareness may trigger eco‐anxiety; however, such anxiety does not always translate directly into environmentally friendly behaviours. In this context, the importance of maintaining climate awareness in a balanced and structured manner in order to prevent eco‐anxiety from reaching undesirable levels has been emphasized (Mat and Yılmaz 2024).

In this study, the scale used was titled as a Carbon Footprint Awareness Scale; however, its items go beyond awareness by addressing environmentally responsible actions and daily practices, including items such as ‘I try to separate waste materials for recycling’. Therefore, the construct assessed by the scale may be interpreted as reflecting both awareness and self‐reported environmentally responsible behaviours. Despite being experienced in different ways among individuals, climate change anxiety has also been described as a potential motivational force for environmentally friendly behaviours in the fight against climate change (Clayton 2020). A growing body of literature indicates significant associations between higher levels of anxiety and increased tendencies toward pro‐environmental behaviours (Clayton and Karazsia 2020; Hickman 2020). Nevertheless, some studies have reported that although university students express concerns about climate change, they may lack sufficient knowledge about the concept and hold certain misconceptions (Li and Liu 2022). In contrast, a consistent trend in the literature indicates that increases in climate change awareness and knowledge are accompanied by parallel increases in climate‐related anxiety and hopelessness (Ediz and Yanik 2023).

Additional subgroup analyses revealed no significant differences in carbon footprint scores according to academic year or place of residence (metropolitan vs. non‐metropolitan). However, previous studies have reported statistically significant differences in carbon footprint scores according to academic year (Moselhy et al. 2022). Similarly, academic stage has been shown to be associated with support for the implementation of environmental measures aimed at reducing carbon footprint (Xu et al. 2025). Furthermore, nurses with higher educational levels are considered more likely to recognize the importance of environmental protection and sustainable healthcare development because of their broader knowledge base and greater educational exposure (Luque‐Alcaraz et al. 2024). In contrast, no differences according to academic year were observed in the present study. This finding may be explained by the absence of carbon footprint‐related courses in the curriculum across all academic years during the study period. Therefore, carbon footprint‐related behaviours may be relatively similar regardless of academic year, previous awareness of carbon footprint, or place of residence. According to the IPCC Sixth Assessment Report, urban areas are among the regions where climate change‐related risks are concentrated, and city residents may experience climate risks more visibly because of air pollution, urban heat island effects, transportation‐related emissions, and infrastructural vulnerabilities (Legg 2021). However, no significant differences according to place of residence were observed in the present study. One possible explanation is that all participants were continuing their education in the same city during the study period, resulting in relatively similar environmental exposures and experiences. In addition, current experiences may have played a more prominent role than past residential experiences in shaping climate change awareness and anxiety.

Within this context, a key question emerges regarding whether the anxiety observed among nursing students stems from a lack of knowledge or from the perceived threat generated by increased awareness and understanding. Notably, the hierarchical regression analysis in the present study demonstrated that the model became statistically significant and that the explained variance increased to 13% with the inclusion of climate change awareness and climate change anxiety in the second model, suggesting that psychosocial variables may contribute to explaining self‐reported carbon footprint awareness‐related behaviours. In particular, the finding that climate change anxiety showed a relatively stronger association than climate change awareness may suggest that environmental behaviours are influenced not only by cognitive processes but also by emotional responses. This may indicate that knowledge about climate change alone is not always sufficient to promote environmentally responsible behaviours and that emotional responses accompanying such knowledge could also play a role. However, alternative explanations should also be considered. Climate‐related anxiety among nursing students may stem not only from increased awareness and understanding but also from media exposure, uncertainty about the future, personal vulnerability perceptions, or broader psychological distress. This may highlight the potential value of climate change education approaches that go beyond information provision and include supportive and structured strategies aimed at helping students cope with emotional responses while encouraging environmentally responsible actions. Therefore, these findings should be interpreted with caution, as the outcome measure may capture environmentally responsible behavioural tendencies in addition to awareness itself, which may partly explain the observed associations with climate change anxiety and awareness.

## 5 Strengths and Limitations of the Work

The present study has several strengths that warrant acknowledgment. A significant strength is the relatively large sample size, which enhances the robustness of the findings and supports the reliability of the statistical analyses conducted. Focusing on nursing students also represents a strength, as this group constitutes future healthcare professionals who will play a critical role in climate change mitigation and sustainability‐oriented practices.

However, several limitations should be considered when interpreting the results. First, the study was conducted with students from a single university located in one geographical region, which may limit the generalizability of the findings to nursing students in other regions or educational contexts. Second, at the time of data collection, the nursing curriculum of the participating institution did not include a specific course related to climate change or sustainability. This may have influenced students' levels of climate change awareness and anxiety, potentially leading to lower exposure to formal education on these topics. In addition, the cross‐sectional design limits the ability to draw causal inferences regarding the relationships between climate change awareness, climate anxiety, and carbon footprint–related behaviours. The use of self‐report measures may also have introduced response and social desirability biases. Furthermore, the relatively low explained variance of the regression model (*R* ^2^ = 0.13) suggests that additional unmeasured factors may contribute to carbon footprint–related behaviours. Another important limitation concerns the measurement tool used in this study, as the Carbon Footprint Awareness Scale includes items reflecting environmentally responsible behaviours in addition to awareness itself. Therefore, the findings should be interpreted cautiously when discussing awareness‐related outcomes.

In addition, the survey link was distributed by faculty members who taught the participating students. Although participation was entirely voluntary, this recruitment approach may have introduced a potential risk of selection bias, which should be considered when interpreting the findings. In addition, previous experiences related to the adverse consequences of climate change may influence students' awareness, anxiety, and behavioural responses. However, these experiences were not assessed in the present study. Future studies may consider including such experiences to provide a more comprehensive understanding of factors associated with climate change awareness and anxiety.

## 6 Recommendations for Further Research

The climate crisis is intensifying worldwide at an increasingly rapid rate. In this context, accurately identifying the predictors of carbon footprint‐related behaviours is crucial for shaping effective and targeted interventions aimed at mitigation. Future studies should investigate additional cognitive, emotional, educational, and contextual factors associated with carbon footprint–related behaviours among nursing students. Determining these predictors will contribute to the development of effective strategies at both individual and societal levels to reduce carbon footprints. Longitudinal and multi‐centre studies are needed to better understand the direction and stability of the observed relationships across different educational settings and populations. In addition, future research should aim to distinguish more clearly between awareness and environmentally responsible behaviours by using measurement tools with stronger conceptual specificity. Simulation‐based educational interventions may also be useful in promoting environmentally responsible behaviours and strengthening climate‐related competencies among nursing students. Lastly, future studies should assess students' previous experiences related to climate change and environmental events, as such experiences may influence climate change awareness, anxiety, and carbon footprint‐related behaviours.

## 7 Implications for Nursing Practice and Policy

The findings of this study have important implications for nursing education, health policy, and clinical practice. To strengthen climate change awareness and promote sustainable behaviours among future nurses, developing a shared core curriculum that addresses climate change, sustainability, carbon footprint literacy, and environmental health across nursing programs should be considered. Integrating standardized content into undergraduate nursing education may help ensure that all students graduate with a minimum and consistent level of knowledge and competence related to climate change and carbon footprint.

In addition, policymakers and educational institutions may consider encouraging nursing students to participate in at least one structured social responsibility or community‐based project related to environmental sustainability prior to graduation and promoting sustainable transportation habits. Furthermore, raising awareness about reducing the use of single‐use materials during clinical education and supporting sustainable practices may contribute to the development of environmentally responsible behaviours.

Furthermore, organizing peer‐led educational initiatives on climate change and sustainability may represent a feasible and effective strategy within nursing programs. Peer education approaches could enhance student engagement, normalize discussions around climate‐related concerns, and support the transformation of climate awareness and anxiety into collective action and professional responsibility.

## Author Contributions

T.Ş.T., B.İ.A., and S.K.Ç. conceived and designed the study. T.Ş.T., B.İ.A., and S.K.Ç. were responsible for data collection. S.K.Ç. performed the statistical analyses, prepared the tables, and drafted the Results section. T.Ş.T. drafted the manuscript. T.Ş.T., B.İ.A., and S.K.Ç. critically revised the manuscript for important intellectual content and approved the final version of the manuscript.

## Funding

The authors have nothing to report.

## Ethics Statement

Ethical approval for this study was granted by the Istanbul Koç University Ethics Committee (approval number 2025.555.IRB3.219, dated 2 December 2025). Throughout the study, strict adherence was maintained to the ethical principles outlined in the Declaration of Helsinki, including confidentiality, beneficence, respect, justice, and non‐maleficence.

## Consent

Written informed consent was obtained from all participants prior to the commencement of the study. Participants were clearly informed that they had the right to withdraw from the study or terminate their participation at any time without penalty. The confidentiality of participant information was one of the highest priorities. All collected data were used solely for research purposes and kept strictly confidential. No personally identifiable information was included in the study reports.

## Conflicts of Interest

The authors declare no conflicts of interest.

## Supporting information


**File S1:** STROBE checklist for reporting cross‐sectional studies.

## Data Availability

The data that support the findings of this study are available on request from the corresponding author. The data are not publicly available due to privacy or ethical restrictions.
